# Soluble Expression of Humanized Anti-CD20 Single Chain Antibody in *Escherichia coli* by Cytoplasmic Chaperones Co-expression

**Published:** 2018

**Authors:** Mohammadreza Yousefi, Safar Farajnia, Ahad Mokhtarzadeh, Bahman Akbari, Shiva Ahdi Khosroshahi, Mina Mamipour, Hassan Dariushnejad, Vahideh Ahmadzadeh

**Affiliations:** 1. Department of Biotechnology, Higher Education Institute of Rab-Rashid, Tabriz, Iran; 2. Drug Applied Research Center, Tabriz University of Medical Sciences, Tabriz, Iran; 3. Faculty of Medicine, Gonabad University of Medical Sciences, Gonabad, Iran; 4. Department of Medical Biotechnology, Faculty of Medicine, Kermanshah University of Medical Sciences, Kermanshah, Iran; 5. Biotechnology Research Center, Tabriz University of Medical Sciences, Tabriz, Iran; 6. Immunology Research Center, Tabriz University of Medical Sciences, Tabriz, Iran

**Keywords:** Molecular chaperones, Non Hodgkin lymphoma, Single chain antibody

## Abstract

**Background:**

CD20 is an important cell surface receptor that is used for target therapy of B cell lymphoma and some related blood diseases due to vital function of CD20. In previous studies, a Rituximab based humanized single chain variable fragment (scFv) antibody showed good reactivity against B cell related cancer cells. But this recombinant protein produced Inclusion Bodies (IBs) in *Escherichia coli (E. coli*) cytoplasm. The aim of this study was to investigate the effect of coexpression with cytoplasmic chaperones on expression and solubility of humanized anti-CD20 scFv in *E. coli*.

**Methods:**

For this purpose, the fragment coding for anti-CD20 huscFv subcloned into the pET22b (+) and transformed into the *E. coli* BL21 (DE3) was evaluated. In order to inhibit the production of IBs, the effects of co-expression with cytoplasmic chaperones GroEL, DnaK, GroES, Tig, DnaJ and GrpE were investigated.

**Result:**

Coexpression with cytoplasmic chaperones led to increased soluble expression of anti-CD20 recombinant protein. Among investigated chaperones, pKJE7 chaperone plasmid containing DnaJ, GrpE, DnaK chaperone genes had significant effects with an expression yield of 325 *μg/ml* soluble anti-CD20 scFv.

**Conclusion:**

The result of this study demonstrated remarkable effect of pKJE7 chaperone on enhancement of soluble expression of anti-CD20 huscFv antibody in *E. coli*.

## Introduction

CD20 is a cell surface marker that is expressed on B cell and presented during the stage of pre B cells. CD20 directly converts the phospholipase C-gamma (PLCγ) to active Ca_2_^+^ transport pump across the plasma membrane 1. Non Hodgkin Lymphoma (NHL) is one of the malignant diseases that is associated with overexpression of CD20 on B cell surface. Hence, anti-CD20 antibodies are approved as an effective treatment method for targeted therapy of B cell malignancies.

Rituximab is an anti-CD20 monoclonal antibody (mAB) 2 that is utilized for treating NHL often culminating in long-lasting response 3. In recent years, various formats of mAb have been developed, such as Fragment antigen-binding (Fab), single chain variable fragments (scFv) and disulfide-stabilized Fv antibody fragment (dsFv). Among them, the scFv format has been extensively studied for various applications 4. Recombinant scFv is generated by joining VH and VL domains through a short polypeptide linker or disulphide bond 5 and *Escherichia coli* (*E. coli*) are generally used as host cells 6–8. IBs are composed of proteins with inappropriate structures. To overcome this problem, various strategies have been developed including the use of molecular and chemical chaperones 9,10.

Molecular chaperones are now well established as cytoplasmic and periplasmic chaperones 11,12. GroEL, DnaK, GroES, Tig, DnaJ and GrpE are major cytoplasmic chaperones in *E. coli*. It has been shown that cytoplasmic chaperones inhibit the rate of Inclusion Bodies (IB) formation leading to increase in solubility of recombinant proteins 13,14. The cytoplasmic chaperones are generally expressed in derivatives of pACYC vector containing chloramphenicol resistance gene (Cmr), araB (ribulokinase) and/or Pzt1 (Tet) promoters which are inducible with L-Arabinose and Tetracycline, respectively. This article is the first report about the application of chaperone plasmids encoding GroEL, DnaJ, Tig, GroES, DnaK and GrpE chaperones to increase the soluble expression of anti-CD20-huscFv in *E. coli*.

## Materials and Methods

### Bacterial strains, media, and plasmids

*E. coli* DH5α, BL21 (DE3) and expression vector pET22b (+) were purchased from Novagen. IPTG, Taq DNA polymerase was obtained from Promega Company. Restriction enzyme and T4 DNA ligase were purchased from Takara Shuzo (Kyoto, Japan). The pUC-57-huscFv construct was obtained from V. Ahmadzadeh 15. The chaperon plasmid set was purchased from TAKARA Bio Inc. (Table 1). Also, the Raji cell line, Ni-NTA resin and protein size marker were obtained from Pasteur Institute of Iran, Qiagen and Fermentas, respectively.

**Table 1.** The detailed characteristics of plasmids used in the present study

**Plasmid**	**Proteins**	**Drug resistance marker**	**Promoter**	**Inducer**	**Reference**
**pUC57**	hscFv-antiCD20	Amp	lac	IPTG	[15]
**pET22b(+)**	hscFv-antiCD20	Amp	T7	IPTG	This study
**pG-KJE8**	dnaK-dnaJ-grpE-groES-groEL	Cm	araB	L-Arabinose	(Takara, Japan)
			Pzt1	Tetracyclin	
**pGro7**	groES-groEL	Cm	araB	L-Arabinose	(Takara, Japan)
**pKjE7**	DnaK-dnaJ-grpE	Cm	araB	L-Arabinose	(Takara, Japan)
**pTf16**	groES-groEL-tig	Cm	Pzt1	Tetracyclin	(Takara, Japan)
**pG-Tf2**	tig	Cm	araB	L-Arabinose	(Takara, Japan)

### Cloning and expression of humanized single chain antibody

The pUC57 containing target gene (pUC57-hsc-Fv construct) was double digested by restriction enzymes MlsI (MscI) and XhoI (at 5′ and 3′ ends, respectively). The insert (huscFv) was isolated and subcloned into the pET22b expression vector containing pelB leader sequence to facilitate expression of recombinant protein in the periplasmic space. For confirmation of this recombinant construct (pET22b-huscFv), PCR was used and restriction analysis and sequencing were carried out.

PET-22b-hscFv construct was transformed to BL21 (DE3) and was grown in LB medium complemented with 100 *μg ml*^−1^ ampicillin. The culture then was incubated with shaking (150 *rpm*) at 25°*C* until culture was induced at A_600_=0.7 using 1 *mM* IPTG for 4, 24 *hr*. Finally, protein expression was analyzed by 12% SDS-PAGE, followed by visualization with Coomassie Brilliant Blue staining and quantitative analysis by using image processing program called ImageJ (National Institute of Health, Bethesda, MD).

### Soluble expression of huscFv by co-expression of pET22b-huscFv with chaperones plasmids

For soluble expression of huscFv, *E. coli* BL21 (DE3) containing pET22b-huscFv bacterial cells were transformed with various chaperones plasmids. Five chaperones including plasmids pG-KJE8, pGro7, pKj-E7, pG-Tf2 and pTf16 were used. The results from these transformations give the BL21(DE3)/pET22b-huscFv/pG-KJE8, BL21(DE3)/pET22b-huscFv/pGro7, BL21(DE3)/pET22b-huscFv/pKjE7, BL21(DE3)/pET2 2b-huscFv/pG-Tf2, BL21(DE3)/pET22b-huscFv/pTf16. The transformed cells were cultured in 100 *ml* LB medium containing 20 *μg/ml* chloramphenicol and 50 *μg/ml* ampicillin. Induction of cells containing chaperones pGro7, pKjE7 and pG-Tf2 was carried out by L-arabinose, pG-KJE8 by L-arabinose and tetracycline, and pTf16 by tetracycline. For expression of chaperones related inducers, L-arabinose 0.6 *mM* for pGro7, pKjE7and pTf16, tetracycline 10 *ng/ml* for pG-Tf2 and both L-arabinose and tetracycline for pG-Tf2 were added into the100 *ml* culture medium in the initiation of culture. When the culture OD reached to 0.7, 0.4 *mM* IPTG was added for induction of huscFv and incubation was continued for 4 *hr* with shaking (150 *rpm*) at 30°*C*. Then, the cells were harvested by centrifugation at 10000 *g* for 3 *min* at room temperature. The cells were resuspended in 10 *ml* of lysis buffer (100 *mM* NaCl, 50 *mM* NaH_2_PO_4_ at pH=8.0) and were disrupted by sonication (five 30 *s* pulses interrupted with cooling on ice).

### Purification of huscFv

For purification of recombinant protein, the harvested cells were sonicated as described above and soluble part of cells was subjected to affinity chromatography. Briefly, supernatant was applied onto a column containing 2 *ml* Ni-NTA resin. The column then washed with buffer A (5 *mM* and 20 *mM* of imidazole) to remove nonspecific proteins. Finally the sample was eluted by elution buffer containing 250 *mM* imidazole. The purified recombinant protein was analyzed by 12% SDS-PAGE followed by staining with Coomassie Blue G250. The concentration of purified huscFv was analyzed by nanodrop analyser.

### Indirect ELISA for antigen-binding activity of anti-CD20 huscFv

To assay the affinity and determine the antigen binding activity of humanized single chain antibody, the Raji cell lysates (10^6^ cells per *ml*) were coated in ELISA plates (96-well plate) and incubated overnight at 4°*C*. After coating, the plates were washed three times with PBS and blocked by 300 *μl* blocking buffer (PBS buffer containing 3% BSA and 0.05% Tween 20) (PBS-T) for 1 *hr* at room temperature. In the next step, serial dilutions of soluble huscFv were added to the wells. After washing with PBS-T, 100 *μ* of HRP conjugated protein L (1:3000) were added into the wells, incubated for 1.5 *hr* at RT, and washed with PBS-T. The reaction was developed with 100 *μ* of TMB at room temperature. Finally, reactions were determined by reading the Optical Density at 450 *nm* (OD_450_) to evaluate the affinity of antibodies.

## Results

### Construction of expression plasmid pET22b-anti-CD20 huscFv

**Figure 1.**
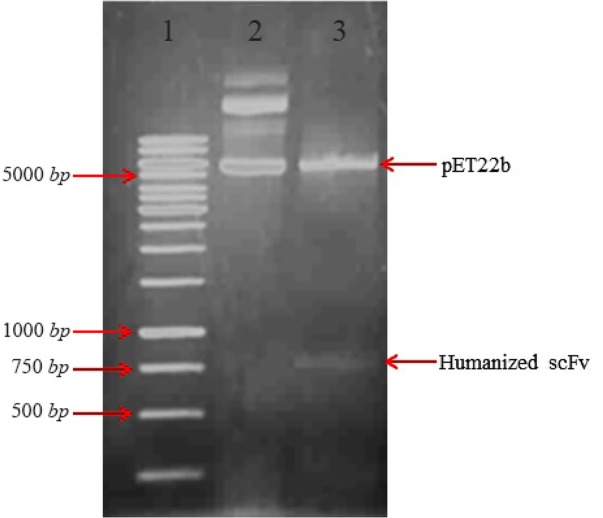
Restriction enzymes digest recombinant plasmid pET22b (+)-huscFv. Lane 1: DNA weight marker. Lane 2: undigested constructs of pET22b-huscFv plasmid by MlsI (MscI) and XhoI restriction enzymes. Lane 3: pET22b-huscFv plasmid after MlsI (MscI) and XhoI double digestion.

For cloning and expression of anti-CD20 huscFv in *E. coli*, the pUC57 plasmid containing the gene coding of huscFv was digested with restriction digestion enzymes MlsI (MscI) and XhoI and the insert was subcloned into the MlsI (MscI) and XhoI site of pET22b (+) expression vector. This subcloning was further confirmed by subsequent restriction digestion analysis (Figure 1).

### Expression of recombinant protein

**Figure 2.**
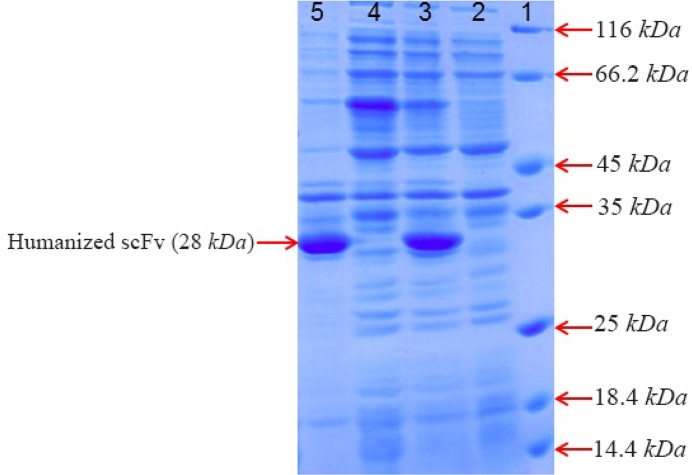
SDS–PAGE analysis of anti-CD20 huscFv recombinant protein expression after induction with 1 *Mm* IPTG for 4, 24 *hr* at 25°*C*. Lane 1: the standard protein weight marker. Lane 2, 4: cell lysate supernatant for 4, 24 *hr*, respectively. Lane 3, 5: insoluble pellet for 4, 24 *hr*, respectively.

For expression of anti-CD20 huscFv, the BL21 (DE 3) containing ET22b-huscFv was cultured in LB broth containing 100 *mg/ml* ampicillin. When the cell concentration reached approximately to the OD 600 of 0.7, the culture was induced by addition of 1 *Mm* IPTG for 4 and 24 *hr* at 25°*C*. Analysis by SDS-PAGE indicated that anti-CD20 huscFv was efficiently expressed in *E. coli* with a band of about 28 *kDa*. Analysis for solubility of recombinant huscFv showed that the majority of huscFv was expressed as IBs (Figure 2).

### Soluble expression of huscFv by co-expression of pET22b-huscFv constructs with chaperones plasmids

**Figure 3.**
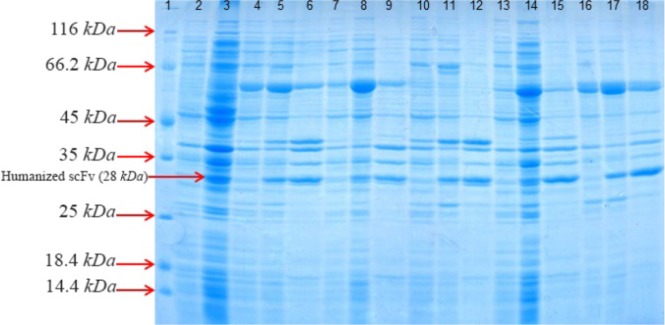
SDS–PAGE analysis of co-expressed anti-CD20 huscFv with different cytoplasmic molecular chaperones after induction with 0.4 *Mm* IPTG for 4 *hr* at 30°*C*, washed and resuspended in lysis buffer 10 *ml* of lysis buffer (100 *mM* NaCl, 50 *mM* NaH2PO4 at pH=8.0). Lane 1: the standard protein weight marker. Lane 2: whole cell lysate supernatant from uninduced cells without chaperone plasmid set. Lane 3: whole insoluble pellet from uninduced cells without chaperone plasmid set. Lanes 4–6: samples of different extracts from *E. coli*BL21 (DE3)/pET22b-huscFv/pG-KJE8 (Lane 4: supernatant from uninduced cells, Lane 5: supernatant from 4 *hr*, Lane 6: pellet from 4 *hr*). Lanes7–9: samples of different extracts from *E. coli* BL21 (DE3)/pET22b-huscFv/pGro7 (Lane 7: supernatant from uninduced cells, Lane 8: supernatant from 4 *hr*, Lane 9: pellet from 4 *hr*). Lanes 10–12: samples of different extracts from *E. coli* BL21 (DE3)/pET22b-huscFv/pKjE7 (Lane 10: supernatant from uninduced cells, Lane 11: supernatant from 4 *hr*, Lane 12: pellet from 4 *hr*). Lanes 13–15: samples of different extracts from *E. coli* BL21 (DE3)/pET22b-huscFv/pG-Tf2 (Lane 13: supernatant from uninduced cells, Lane 14: supernatant from 4 *hr*, Lane 15: pellet from 4 *hr*). Lanes 16–18: samples of different extracts from *E. coli* BL21 (DE3)/pET22b-husc Fv/pTf16 (Lane 16: supernatant from uninduced cells, Lane 17: supernatant from 4 *hr*, Lane 18: pellet from 4 *hr*).

In order to investigate the contribution of different molecular chaperones in soluble expression of anti-CD20 huscFv in *E. coli* BL21 (DE3), this anti-CD20 huscFv was co-expressed with different chaperones plasmids. The result indicates that chaperones enhanced soluble expression of huscFv. Co-expression of the huscFv-anti-CD20 with chaperone plasmid sets including pGro7, pG-KJE8, pTf16, pKjE7 and pG-Tf2 indicated enhancement in solubility in comparison to anti-CD20 huscFv that was expressed without the chaperones and the result was analyzed by NanoDrop which is shown in table 2. Importantly, the co-expression of pKJE7 containing GrpE/DnaK/DnaJ has the highest outcome (up to 50%) on soluble expression of recombinant huscFv compared to pGro7, pG-KJE8, pTf16 and pG-Tf2 chaperons (Figure 3).

**Table 2.** Comparison of plasmids relative activity with different expression levels of huscFv

**Plasmid**	**Concentration of insoluble huscFv (*μg/ml*)**	**Concentration of soluble huscFv (*μg/ml*)**	**The quantity of resultant refolded huscFv (*μg*)**	**The ratio of solubility in total huscFv (%)**	**The ratio of insolubility in total huscFv (%)**
**huscFv-antiCD20**	43.15	16.4	82	27.53	72.47
**huscFv + pG-KJE8**	64.12	51.31	256.5	44.45	55.55
**huscFv + pG-Tf2**	76.47	42.76	213.8	35.86	64.14
**huscFv + pKJE7**	76.51	65.13	325.65	45.98	54.02
**huscFv + pGro7**	52.41	34.26	171.3	39.52	60.48
**huscFv + pTf16**	53.70	27.2	136	33.62	66.38

### Purification of huscFv

**Figure 4.**
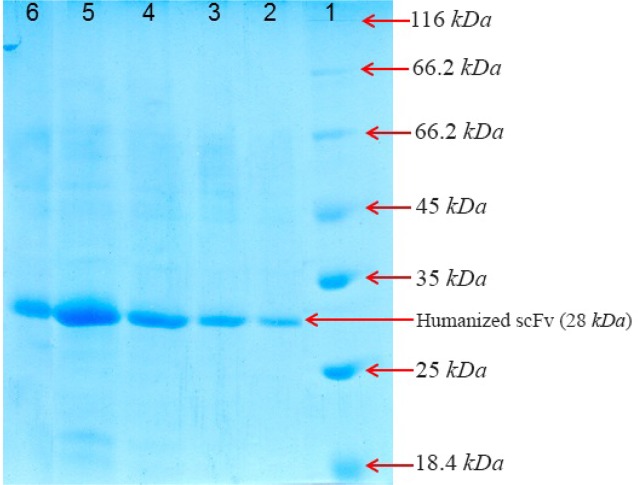
SDS-PAGE analysis of the purified recombinant huscFv in BL21 (DE3) after gel filtration chromatography and washing with buffer A (5 *mM* and 20 *mM* of imidazole). Lane 1: the standard protein weight marker Lane 2: purified huscFv/pTf16 Lane 3: purified huscFv/pGro7 Lane 4: purified huscFv/pG-KJE8 Lane 5: purified huscFv/pKjE7 Lane 6: purified huscFv/pG-Tf2

After cell harvest and lysis by sonication of the cells, soluble fractions were collected by centrifugation at 10000 *g* for 10 *min* and recombinant protein was purified by affinity chromatography method from supernatant of *E. coli* cell lysate. The result showed that purity of the recombinant protein was in high level and determined by SDS-PAGE and seemed as a single band (Figure 4). Afterwards, the concentration of refolded huscFv protein was determined by NanoDrop which is shown in table 2.

### ELISA result

ELISA experiments were accomplished with anti-CD20-huscFv.The results demonstrated that huscFv is refolded by pKJE7, pG-KJE8 and pG-Tf2 chaperones had higher binding activity compared to huscFv without chaperone co-expression. On the other hand, the relative ELISA results of huscFv refolded by pGro7 and pTf16 chaperones are similar to huscFv without chaperone co-expression. All of the chaperones, husc-Fv refolded by pKJE7, revealed the highest binding activity.

## Discussion

CD20 antigen expressed on cell surface of immature B cells is a validated target for treatment by anti CD20 mAb, Rituximab. This mAb is a chimeric monoclonal antibody and is approved for treatment of B-cell Non-Hodgkin’s Lymphoma (NHL) disease 16.

Due to the size and cost related drawbacks of full length monoclonal antibodies, development of smaller versions of these antibodies using low cost expression system of *E. coli* is the subject of great interest 17. In spite of low cost and high yield of protein production in *E. coli*, this expression system suffers from several disadvantages. Expression of eukaryotic proteins in *E. coli* generally results in production of the recombinant proteins as inclusion body that lack correct 3D structure 18. IBs are composed of proteins with inappropriate structures that are the result of protein expression in non-physiological conditions in the cytoplasm of *E. coli*. Periplasmic expression is one of strategies to overcome this impediment. Hence, pelB leader peptides were used to allow translocation of unfolded recombinant protein to the oxidizing space of periplasm. The disulphide bonds have an important role in correct folding of recombinant proteins that lead to high stability. So, productions of recombinant proteins in soluble form are the subject of extensive researches. For increasing soluble expression of anti-CD20-huscFv, various approaches are used including co-expression with molecular chaperone, use of chemical chaperones and optimization of the culture conditions. The present study aimed to evaluate the effects of co-expression with different chaperons on soluble expression of recombinant anti-CD20 huscFv 9,19,20. For this purpose, the sequence encoding for Rituximab based hscFv was transformed into the BL21 (DE3) for expression.

In our study, partial soluble expression of the husc-Fv was observed without using any molecular chaperones. Hu X *et al* indicated that the use of low temperature and low rate of inducer concentration led to enhancement in correct folding and finally increased concentration of soluble and functional form of recombinant proteins 21.

On the other hand, for enhancement of the huscFv soluble expression, various plasmids containing chaperone combinations have been used that revealed remarkable enhancement (up to 50%) of soluble expression in the cytoplasm. The related studies are in agreement with our results 22,23. Among different chaperone plasmids checked by co-expression, the pKJE7 plasmid set containing DnaK, DnaJ, GrpE chaperone genes demonstrated higher soluble yield in comparison to pGro-7, pG-KJE8, pTf16 and pG-Tf2 plasmids. High level of expression of huscFv with pKJE7 plasmid set depended on close activation between DnaK, DnaJ, GrpE chaperone proteins. The results were in agreement with studies done by Bo *et al*. They reported that anti-BNP scFv was expressed in soluble form by using pKJE7 plasmid set that has the highest amount of scFv, while increasing expressed soluble form of anti-BNP scFv led to reduction of anti-BNP scFv expression 23. In another study, Heo *et al* reported the enhancement of soluble expression of anti-c-Met scFv with co-expression of cytoplasmic chaperones 24. On the contrary, Nishihara *et al* showed that chaperone proteins containing GroEL and GroES have synergic effect with GrpE, DnaK, DnaJ in helping folding an Allergen of Japanese Cedar Pollen, Cryj2, in *E. coli* 19.

However, in our study, there were not any relative synergistic effects between GroEL and GroES proteins with GrpE, DnaK, DnaJ chaperone proteins. Also, in this study, binding activity of soluble huscFv produced by co-expression of combination of various cytoplasmic chaperones was analyzed by relative ELISA. The result showed that all of the soluble fractions had positive effects in binding activity, among them co-expression with plasmid pKJE7 led to 50% increase in husc-Fv binding activity compared with and without chaperone coexpression. Increasing the binding activity of huscFv co-expressed with plasmid pKJE7 depended on the absence of both GroELS and DnaKJE. These results indicated that there is not any constructive collaboration between GroELS and DnaKJE. Similar results were previously reported for recombinant protein production in the cytoplasm. Sonoda H *et al* proved that co-expression of DnaKJE with GroELS has negated the effects of GroELS 25. Furthermore, Sonoda H *et al* proved that in the cytoplasmic production system, co-expression of GroES/GroEL displays 4.6 fold evaluation in antigen-binding activity 26.

## Conclusion

The result of this study indicates that co-expression of pKJE7 cytoplasmic chaperone with huscFv leads to 50% increase in soluble expression. Furthermore, the result of binding activity revealed that all chaperon plasmids had positive effect in huscFv binding activity. Among them, the maximum huscFv binding activity was obtained when huscFv was co-expressed with pKJE7 plasmid chaperone set.
